# Amino acid fermentation at the origin of the genetic code

**DOI:** 10.1186/1745-6150-7-6

**Published:** 2012-02-10

**Authors:** Harold P de Vladar

**Affiliations:** 1 Am Campus 1. Klosterneuburg A3400. Austria

## Abstract

There is evidence that the genetic code was established prior to the existence of proteins, when metabolism was powered by ribozymes. Also, early proto-organisms had to rely on simple anaerobic bioenergetic processes. In this work I propose that amino acid fermentation powered metabolism in the RNA world, and that this was facilitated by proto-adapters, the precursors of the tRNAs. Amino acids were used as carbon sources rather than as catalytic or structural elements. In modern bacteria, amino acid fermentation is known as the Stickland reaction. This pathway involves two amino acids: the first undergoes oxidative deamination, and the second acts as an electron acceptor through reductive deamination. This redox reaction results in two keto acids that are employed to synthesise ATP via substrate-level phosphorylation. The Stickland reaction is the basic bioenergetic pathway of some bacteria of the genus *Clostridium*. Two other facts support Stickland fermentation in the RNA world. First, several Stickland amino acid pairs are synthesised in abiotic amino acid synthesis. This suggests that amino acids that could be used as an energy substrate were freely available. Second, anticodons that have complementary sequences often correspond to amino acids that form Stickland pairs. The main hypothesis of this paper is that pairs of complementary proto-adapters were assigned to Stickland amino acids pairs. There are signatures of this hypothesis in the genetic code. Furthermore, it is argued that the proto-adapters formed double strands that brought amino acid pairs into proximity to facilitate their mutual redox reaction, structurally constraining the anticodon pairs that are assigned to these amino acid pairs. Significance tests which randomise the code are performed to study the extent of the variability of the energetic (ATP) yield. Random assignments can lead to a substantial yield of ATP and maintain enough variability, thus selection can act and refine the assignments into a proto-code that optimises the energetic yield. Monte Carlo simulations are performed to evaluate the establishment of these simple proto-codes, based on amino acid substitutions and codon swapping. In all cases, donor amino acids are assigned to anticodons composed of U+G, and have low redundancy (1-2 codons), whereas acceptor amino acids are assigned to the the remaining codons. These bioenergetic and structural constraints allow for a metabolic role for amino acids before their co-option as catalyst cofactors. Reviewers: this article was reviewed by Prof. William Martin, Prof. Eörs Szathmáry (nominated by Dr. Gáspár Jékely) and Dr. Ádám Kun (nominated by Dr. Sandor Pongor)

## Background

**Figure 1:**
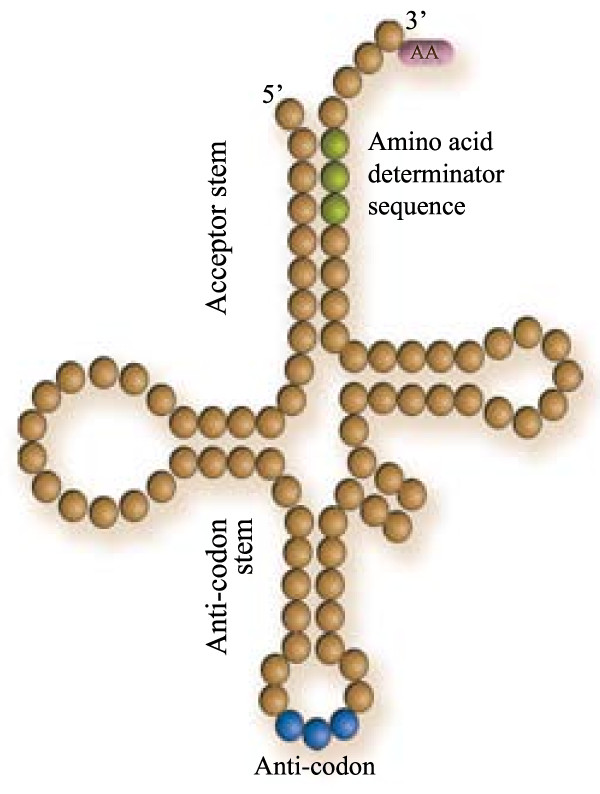
**Basic structure of the tRNA molecule**.

**Figure 2:**
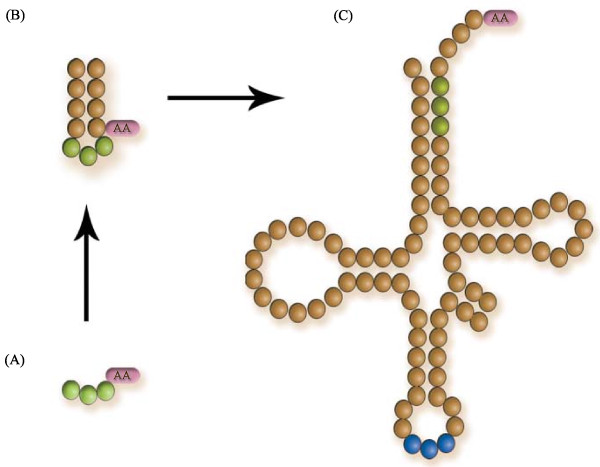
**Stages of the evolution of the tRNA molecule**. (A) Originally this consisted on a trinucleotide attached to an amino acid; (B) the triplet elongated to a mini-helix, probably because it conferred structural stability; (C) further elongations of the mini-helix resulted on the modern tRNA structure. This work is set in a stage as in (B). For details on the actual mechanisms of the evolution of the tRNA molecule, see [6].

The RNA world is an ancient evolutionary period characterised by a ribozyme-based metabolism. It is thought that the genetic code, or at least the precursors to the modern adapters (i.e. tRNAs, Figures 1 and 2), were established at that stage [[1], Ch. 5]. There are several theories that explain the current organisation of the code in terms of selective advantages, robustness against mutation, lateral gene transfer, biochemical and physical-chemical properties, and so forth. Most of these ideas build from the assumption that earlier, perhaps less accurate or complex codes existed, which evolved by selecting on different organisational principles, leading to the modern genetic code. Whilst most theories deal with the rearrangements to the code, only few directly address the question of how this code emerged. To some extent, the specific association between some amino acids and their codons can be explained by the formation of covalent complexes of dinucleotides and precursors to amino acids [2]. Other ideas that have received more attention, are the stereochemical arguments (the structural affinity between amino acids and coding triplets) [3-6]. Furthermore, it has been proposed that the role of amino acids in an RNA world was to improve the catalytic activity of ribozymes [7], a function that requires coding, because ribozymes needed to bind the amino acid as cofactors in a specific way.

I propose that an alternative ancient function for the amino acids in an earlier RNA world was to harvest energy. I propose a scenario in which coding is required in order to carry out the catabolic degradation of amino acids, and which may have easily arisen from initial random assignments.

**Figure 3:**
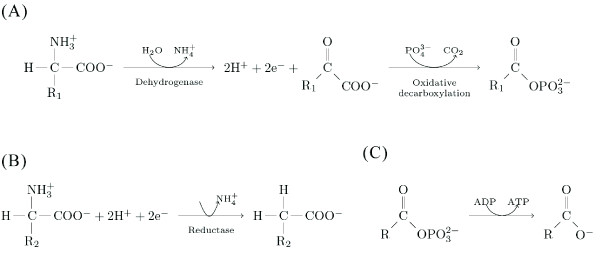
**The Stickland reaction**. In the genus *Clostridium*, amino acid fermentation requires two steps. (A) Oxidative deamination of the amino acid with the higher oxidation state yields an *α*-keto acid, ammonium, and two protons. For example, if R_1 _is a methyl group (-CH_3_), then the donor amino acid is an alanine, and the product of the oxidative deamination would be pyruvate, a central compounds of the intermediary metabolism. (B) Inorganic phosphate attaches at the carboxyl terminus. The second proton takes the place of the amino radical. The product of this reaction is an acyl-phosphate. For example when R_2 _is a hydrogen (thus the acceptor amino acid is a glycine) this product corresponds to acetyl-phosphate. The two phosphate compounds from A and B are substrates for the synthesis of ATP from ADP. Naturally, in *Clostridium spp*. all these reactions occur with the aid of hydrogenases (for the electron donors) and reductases (for the electron acceptors). ATP synthesis is catalysed by kinases. However, in chemo-autotrophic systems, the acetyl-phosphate is readily the energy carrier (see text).

My argument follows from three observations. First, amino acids are catabolised to obtain energy, and the metabolites are used as precursors for other biomolecules, a role often overlooked due to the prominent and central position of proteins in metabolism. This suggests that the amino acids could have originally had a bioenergetic, rather than catalytic role, with the latter appearing later. In particular, bacteria of the genus *Clostridium *are known to directly use amino acids to harvest metabolic energy. Some of these obligate anaerobes do not employ glucose as a carbon source, but rather ferment a pair of molecules; one amino acid acts as an electron donor and another acts as an electron acceptor. The overall reaction, termed *Stickland fermentation *releases energy the that powers the production of ATP (Figure 3). The substrates for the Stickland reaction are specific pairs of amino acids. That is, the reactants need to include one amino acid that can be oxidised (typically alanine, valine, leucine, serine, isoleucine or threonine), and another amino acid that can be reduced (typically glycine, proline, or aspartic acid; see Table 1) [8,9].

**Table 1:** Amino acids involved in Stickland fermentation in *Clostridium spp.*

e^- ^Donors (reductants)	e^- ^Acceptors (oxidants)	Donors or acceptors (depending on partners)
Alanine* [39,70]	Glycine* [39,42,70]	Leucine*
Valine* [39]	*Aspartic acid*^• ^[9]	Phenylalanine* [9]
Serine* [8,30,42]	Proline^• ^[70]	Tyrosine* [9]
Isoleuine* [30]		Tryptophan* [9]
Threonine^• ^[8]		Arginine^• ^[9]
Glutamic acid^• ^[8]		
Histidine* [8]		
Cysteine		
Methionine		
Lysine		

The underlined compounds are those abiotically synthesised, and the italicised items are the ones included in the "primordial" cycle, Figure 7D. Amino acids marked with '*' are synthesised via the glycolitic (including the pentose phosphate) pathway, and those marked with '•' are synthesised via the citric acid cycle (either oxaloacetate or *α*-ketoglutarate).

Secondly, amino acids are readily produced under numerous potential scenarios for the abiotic synthesis of the building blocks of life. The famous Miller experiment [10] and other Miller-Urey syntheses [11-13] employ energy sources to generate amino acids from gases. The "iron-sulfur" chemo-autotrophic theory [14], which is compatible with the conditions of black-smoker hydrothermal vents (extremely hot and highly acidic), employs the reductive power of sulphides and high temperatures to synthesise organic molecules, including amino acids, via thio esters. In the warm but alkaline hydrothermal vents (the white smokers) a similar reduction of CO_2 _by sulphides occurs, and the reactions that take place are analogous to the acetogenesis (Wood-Ljungdahl) pathway, where the nitrogen-fixation branches lead to amino acids, which are, in turn, also used as precursors to synthesise nucleotides [15].

**Figure 4:**
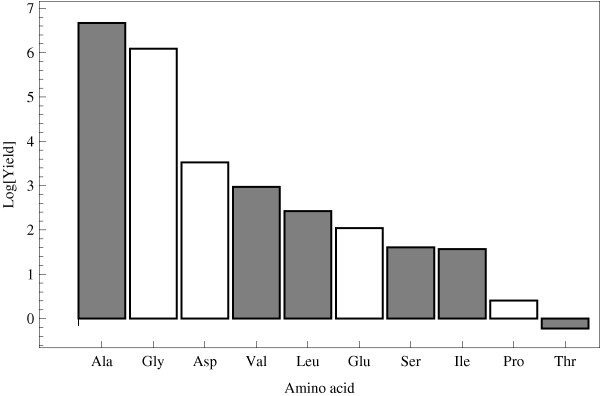
**Yield of amino acids in the Miller experiment**. Yield is measured in *μ*mol; products result from sparkling 336 mmol of methane, which correspond to a total of 1.55%. Data from Table 1 of [13]. The grey and white bars represent amino acids that are electron donors and acceptors in the Stickland reaction, respectively. The yield axis is in a logarithmic scale.

In all three scenarios several "Stickland pairs" are readily formed. In particular, the "Miller amino acids" [10] with the highest yield (principally alanine, glycine, aspartic acid and valine; Figure 4) can form four Stickland pairs: alanine+glycine, alanine+aspartic acid, valine+glycine and valine+aspartic acid. Similarly, in the iron-sulfur world, glycine, serine and aspartic acid are readily synthesised [16], where we find that glycine+serine and glycine+aspartic acid are Stickland-reactive pairs. Finally, besides glycine and alanine, aspartic and glutamic acids are also formed in the alkaline hydrothermal vents [15], again forming the pairs alanine+glycine and alanine+aspartic acid and aspartic acid+alanine.

The third observation is the crucial one; anticodons that are complementary often code for amino acids that have conjugated Stickland pairs. This is particularly true for the "Miller" amino acids (which are the simplest ones), and also holds for complementary anticodons that involve non-canonical G-U pairs. The correlation between these two forms of complementation (the metabolic or Stickland complementation, and the anticodon complementation) is relevant because in the evolution of the proto-adapters, RNA complementation played a role in diversifying the anticodons [6,17,18].

These three independent facts suggest that amino acid fermentation could have played a role in the establishment of the genetic code. The purpose of this article is to develop this idea in more detail, to state the hypothesis in a testable way, and to analyse the implications that it has on our current understanding about the context of the early metabolism and on the factors that established the translational machinery.

### Abiogenesis of amino acids

In the 1920's Oparin [19] and Haldane [20] proposed that under anoxic conditions and with suitable energy sources, organic matter would spontaneously form, which would be the basis for the first forms of life. Later, Miller [10] synthesised amino acids from a mix of four elementary gases: ammonia (NH_3_), methane (CH_4_), water (H_2_O), and carbon dioxide (CO_2_). This mixture forms a "reductive atmosphere", which means that the compounds that are formed have a significant potential to undergo redox reactions. Some compounds are good electron donors, such as ammonia and methane. Carbon dioxide and molecular oxygen are powerful electron acceptors. The presence of electron acceptors is critical for organisms, since they allow the oxidation of carbon sources (e.g. glucose) to water and carbon dioxide, and the concomitant release of free energy is employed for vital metabolic processes. However, contemporary understanding suggests that methane and ammonia were absent from the early Hadean atmosphere (about 4 billion years ago). Instead, molecular nitrogen (N_2_) was abundant [21]. In this "weakly reducing" atmosphere N_2 _is a poor electron donor, which limits both the diversity and the yield of the compounds that can be abiotically formed.

In any case, applying energy to these reducing mixtures synthesises amino acids that are biologically significant. This is true for several of the variants of Miller's experiment [11]. Miller-like experiments usually give relatively high yields of glycine, aspartic acid, alanine and valine, amongst others, depending on the energy source and initial mix [11-13,22]. Figure 4 depicts the relative yields of the reducing atmosphere, in which some important amino acids are readily synthesised.

It is debatable however, under which atmospheric conditions the origin of the genetic code occurred, and therefore which were the amino acids that were relevant for an early metabolism. One possibility is that the genetic code was established just after the origin of life, in a pre-RNA world in a prebiotic-soup setting, under a weakly reducing atmosphere with low yields of glycine, aspartic acid and alanine [11]. This is an unlikely possibility because an abiotic metabolism which included nucleic acids had to exist. The geological conditions during this period were also very harsh, characterized by hostile volcanic activity and high temperatures. These conditions are thought to be too adverse for the establishment of early forms of life, although it is not clear whether autotrophic metabolisms could have actually existed. Another possibility, is that the genetic code was established in a late RNA world, after the atmosphere had already changed, perhaps to include methane and ammonia produced from the metabolism of methanogenic protobionts. This, as we understand it, happened during the Archaean period, about 3.5 billion years ago. This is a more likely scenario because in an already existing metabolism, an emerging code would result from the reorganisation of established processes. In other words, the pre-adaptations that were required for the genetic code to be established are consistent with early metabolisms.

Wächtershäuser summarised and pointed out several reasons why Miller's "prebiotic soup" does not work as a model for prebiotic evolution [14]. Although these reasons are debatable, it is worth considering that a prebiotic soup may in fact not be the most adequate scenario. One strong argument is that the composition of the primitive atmosphere [21] would not consist of the gases needed to synthesise primordial amino acids with enough yield. As an alternative, Wächtershäuser proposed what has been called the "primitive pizza" [1, pp. 32-33]. This theory posits the prebiotic reactions occurring not in solution, but on the surface of pyrites, minerals rich in iron. Ferrous cations (positively charged) and sulphur anions (negatively charged) are adsorbed in the mineral surface and react to form pyrite. This reaction, i.e. Fe^2+ ^+ 2H_2_S →FeS_2 _+ 4H^+ ^+ 2e^-^, liberates two electrons and is exergonic, making energy readily available for other chemical reactions to happen. Amongst the possible chemical pathways, the surface reactions can form amino acids. Most amino acids could in principle be formed. However, the first amino acids that would be formed on pyrite surfaces, and which by tentative implication would be the first to be included in an ancient genetic code, would be serine and aspartic acid. Serine would be immediately cleaved to produce glycine [14]. In experimental syntheses of Wächtershäuser's theory, some amino acids were actually obtained: glycine, alanine and serine [16], and alanine, glutamic acid, phenylalanine, and tyrosine [23,24].

Another chemo-autotrophic scenario that is worth discussing actually occurs at the alkaline hydrothermal vents [15]. Arguably, this setting comprises the most comprehensive view of the origin of energy metabolism. Geosynthesis in the Earth's crust powers serpentinization: silicates of magnesium and iron are oxidised by water and produce H_2_. This molecular hydrogen is later released at the hydrothermal vents, where it reacts with CO_2 _and iron sulphide (FeS), forming thio esters. These compounds are the basis for the synthesis of many compounds and cofactors, and also couple the activation of acetate with phosphate to give acetyl-phosphate, which can be further hydrolysed to end up in acetate (CH_3_COOH). As in the iron-sulfur scenario, FeS can also catalyse the oxidative amination of *α*-keto acids, resulting in alanine, glycine, and aspartic and glutamic acids. Notably, purines and pyrimidines can also be formed from aspartic acid and glycine (a pathway that involves acyl-phosphates) [25].

In summary, these different scenarios provide contextual frameworks that support the synthesis of amino acids. The question of the yield amino acid production still stands; even if these theories reveal plausible pathways for *de novo *amino acid synthesis, experimental assessments have shown that the yield is rather poor compared to the requirements of modern organisms. But was the relatively low abiotic yield of amino acids enough to sustain protobionts based on RNA metabolisms? The answer to that question strongly depends on the role of amino acids at the moment when the genetic code was established.

### The Stickland reaction

The role of the amino acids is inevitably associated with the structural and catalytic nature of proteins. Given their small size, it would be absurd to consider that free amino acids could have a structural role. But because of their physical-chemical properties, free amino acids associated with RNA adaptamers could aid catalysis [7]. The theory of the coding coenzymatic handles (CCH) argues that the amino acids were covalently attached to trinucleotides, in a way that is reminiscent of metabolic cofactors, such as nicotinamide adenine dinucleotide (NADH) or ATP for example [7,26]. The trinucleotides served as "handles" through which ribozymes non-covalently attached to the amino acids. In this way, the amino acids could be re-used to aid catalysis [27]. Any unambiguous association between triplets and the amino acid repertoire would ensure that correct catalytic factors would be used for specific functions. This assumes that a modestly-varied repertoire of amino acids was already available. The synthesis of most catalytically important amino acids is very elaborate, and their abiotic yield is negligible (except for aspartic acid), a fact that necessarily postpones the catalytic functions of the amino acids to later historical stages.

Another fundamental role of protein and amino acid metabolism is nutrition. Amino acids are oxidised via the citric acid cycle and converted into urea (which is disposed of) and pyruvate and other keto acids of the citric acid cycle which are used in the anabolic synthesis of other compounds. Amino acids have a similar oxidation state to that of glucose, suggesting that they can undergo fermentation, and in extant organisms their breakdown fosters ATP synthesis.

There are several catabolic pathways of amino acid degradation. Amongst these, the Stickland reaction is the most efficient, by coupling the oxidation of one amino acid with the reduction of another. The amino acids that are oxidised undergo a deamination by the amino acid dehydrogenases, losing in addition two electrons and two protons [28-30]; the resulting keto acid then loses one carbon to CO_2_, and leads to ATP synthesis by substrate-level phosphorylation (Figure 3A) [9,29-31]. This reaction is then coupled to the "reductive branch" by transferring the electrons and the protons to the other amino acid, which undergoes a reductive deamination, catalysed by a reductase [32-35]. The reduced keto acid has the same number of carbons as the acceptor amino acid. Normally, the reductive branch does not lead to ATP (unless both substrates are glycine), (Figure 3B) [36].

The overall amount of free energy varies according to the specific pair of amino acids undergoing fermentation, and to the stoichiometry of the reaction. For example, fermenting four moles of glycine leads to 3 moles of ATP; fermenting glycine and alanine (in a stoichiometric ratio of 3:1 moles) leads to 1.7 moles of ATP. Both result in lower yield than the fermentation of two moles of glucose, which leads to 5 moles of ATP, but still efficient when compared with the yield of fermenting other carbohydrates (e.g. 3 moles of lactate gives a yield of 2.3 moles of ATP) [[36,37], Ch. 12].

The energetic yield is in fact sufficient to sustain organisms that employ exclusively amino acids as energy sources. The most compelling example is the genus *Clostridium *which comprises chemoautotrophic, anaerobic, bacteria [38]. Various species may or must use amino acids as a carbon source [9,30,35,39,40]. However the genus *Clostridium *is not a monophyletic group [40,41], which indicates that the Stickland reaction may have independent origins (an unlikely possibility because of the sequence similarity of the dehydrogenates and reductases [32]), has been lost in other genera, or was acquired by lateral transfer. Amongst the species we find thermophilic and alkaliphilic, some of which are associated with hydrothermal vents [37,38,40,42,43]. This hardly places the extant Stickland reaction pathways in a deep ancestral branch of the tree of life, but it is conceivable that analogous, if not ancestral, versions of Stickland reaction pathways not only existed before the code was established, but could also have played a role in the establishment of the code, before the catalytic role was implemented and translation evolved.

### Evolution of the adapters

The problem of the usage of amino acids is only one side of the coin. The other side is how amino acids were assigned to coding triplets. Even if the role of amino acids were taken for granted, as with CCH or the Stickland fermentation, the question remains as to which factors determined such assignments. In any case, it can be assumed that at some stage the assignment involved a covalent bond between the amino acid and the proto-adapters. It has been debated whether the handles composed ancient codons or anticodons [5,26], but stereochemical associations favour the idea that these were anticodons [5,6]. These co-enzymatic handles later elongated to form mini-helices. Helical structures could have conferred structural stability to the proto-adapters (tRNA precursors), possibly for coding functions. The ideas introduced in this article assume a mini-helical stage for the proto-adapters. The mini-helices eventually evolved by successive and recursive duplications to form the modern adapters with their cloverleaf structure, the tRNAs (Figure 2, [6]). This idea is founded in a recently proposed mechanism. It argues that the ancestor to the adapters was a primordial palindrome gene composed of 11 nucleotides. This small hairpin would be composed of two block sequences: a coding triplet (the CCH, say, GCC), and a replication tag (something like a promoter sequence) with sequence 5'-DCCA-3' (where D is either an A or a U). If the pre-coding triplet is linked between the tag and the tag's complementary sequence, a small hairpin of 11 nucleotides is formed: UGGDGCCdCCA (where d denotes the complement of D). Now, the tag plus triplet (7 nucleotides) is complementary to the 11 nucleotides block, and can be ligated at one of its ends and form an 18 nucleotides hairpin. If this pattern is iterated, then the 76 nucleotides long molecule, the tRNA, can be built [6]. Notably, in each iteration of this duplication-elongation mechanism two proto-adapter molecules are co-evolving (one in each strand), which supports the long-standing hypothesis that amino acids were included in the code in pairs [6,18,44-47].

**Figure 5:**
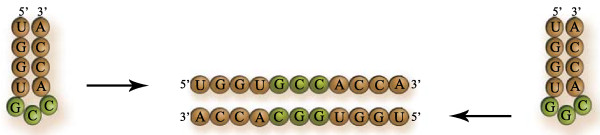
**Primordial palindrome gene**. Two mini-helices that have complementary anticodons but are otherwise identical, can unfold and dimerize to form a RNA double helix. This double-helix is a palindrome.

Because a hairpin has complementary sequence at both strands, then a pair of proto-adapters with complementary anticodons are fully complementary. Furthermore, this RNA double helix must have been a palindrome (see Figure 5). What is striking is that modern pairs of tRNAs with complementary anticodons are also complementary at the amino acid determiner sequences at the stem [6,18,45,46]. This suggests that the sequences at the stem must have corresponded to the precursors of an anticodon. This correlation is stronger when the non-canonical pair G-U is allowed in the helices [45,48,49]. The implication, as detailed below, is that the redundancy of the code can be explained in terms of the coevolution of the anticodon precursors [18,45,46]. First, it is assumed that proto-adapters with complementary anticodons when replicating, form intermediary double stranded helices. Low-fidelity RNA replicators produced unassigned anticodons, perhaps by mutations, but mainly by employing G-U pairing [6]. Second, these unassigned codons were assigned to new amino acids whilst their complements, being similar to the original proto-adapter, were assigned to the original amino acids [18,45]. Consider for example GCC, an anticodon assigned to glycine. This codon can produce an initially unassigned triplet by template replication: GGU. The latter, through proper Watson-Crick matching, produces ACC, which is another anticodon for glycine. The intermediate anticodon GGU was then assigned to a new amino acid (in this case, threonine). This mechanism can be extended to other anticodons, and parsimoniously explain the incorporation of several amino acids in pairs, although it does not explain why specific pairs (say, glycine and threonine) were assigned to the pair of anticodons ACC and GGU.

## Presentation of the hypothesis

**Figure 6:**
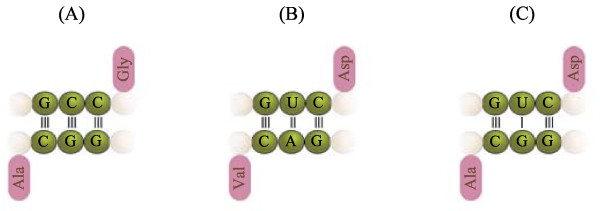
**Pairs of tRNAs with complementary anticodons bearing Stickland-reactive amino acid pairs**. (Only the anticodon sequences shown). The three associations shown correspond to both anticodon complementarities, and Stickland electron donor/acceptor pairs of amino acids: (A) Glycine is an electron acceptor, whilst alanine is a donor. (B) Aspartic acid is an electron acceptor in the presence of valine and (C) also on the presence of alanine. (D) A different codon for glycine associates with one from serine (an electron donor). (A), (B) and (D) are legitimate pairs, in the sense that the anticodon bases match according to Watson-Crick pairing. (C) is an illegitimate association, since it involves the U-G pair. The lines represent the pair types: three for G-C pairs, two for A-U pairs and one for U-G pairs. Gly: glycine, Asp: aspartic acid Ala: alanine, Ser: serine.

The coevolution of the anticodon precursors as described above raises the following question: which amino acids were assigned to the new anticodons? To answer this question, we need to invoke a function for the proto-adapters. A catalytic role for the amino acids cannot be excluded, given that these are compounds with versatile chemical and structural properties. However, there is a gap between the stage when CCH were used as catalytic cofactors and the use of adaptors having a proto-code. What I propose is that the free anticodons were assigned to amino acids that complemented the Stickland role of the amino acids readily assigned to the complementary anticodons. This rule should apply for both legitimate and illegitimate complements (i.e., those involving G-U pairs). For example, in Figure 6 the anticodon GCC for glycine is complementary with the anticodon GGC for alanine. Glycine and alanine are Stickland pairs. Another pair would be formed between GUC (for aspartic acid) and GAC (for valine) where the amino acids are Stickland pairs. However, the illegitimate pair between GUC and GGC can also be formed. In fact, their amino acids, aspartic acid and alanine respectively, are also Stickland pairs. Table 1 lists the Stickland roles of some amino acids, including the ones appearing in the cycle (all of which are "Milller" amino acids, Figure 4). This observation leads to the following hypothesis:

*Complementary anticodons are assigned to amino acids that are conjugated Stickland pairs*.

In other words, the suggestion is that the origin of the genetic code traces back to the mutual redox deamination of the amino acid pairs to synthesise high-energy intermediates, such as acetyl phosphate and other related phosphate compounds.

## Testing the hypothesis

The association between Stickland pairs and complementary anticodons is expected to hold more strongly for the primordial amino acids. Later additions could have been affected by other factors, especially when the bioenergetic pathways had already evolved, and departed from amino acid fermentation.

**Figure 7:**
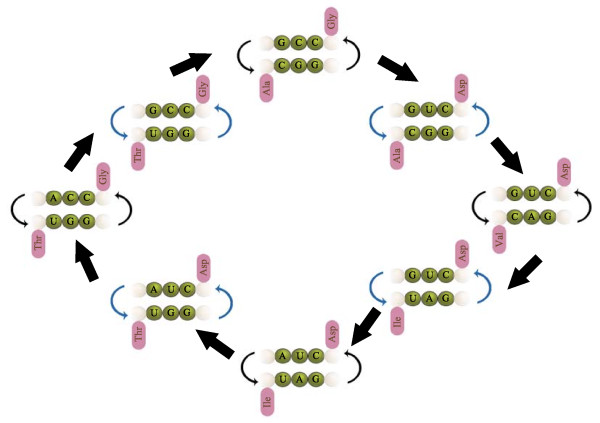
**Cross-catalytic cycle of proto-adapters**. (Only the anticodon sequences shown). Templates with complementary anticodons catalyse each other following Watson-Crick pairing (thin black arrows). However, allowing for non-legitimate pairs G-U allows for cross-catalysis of other templates (thin blue arrows). In this way, each pair of replicators catalyse another cycle of replicators (thick black arrows). This pattern allows formation of closed cycles. This figure shows only one of the possible nested cycles that emerge allowing for non-legitimate template replications. In this cycle, all pairs of templates bear amino acids that are Stickland pairs, and all amino acids are readily formed in Miller-type experiments. Other cycles that involve amino acids that are thought to be included at later evolutionary stages can show exceptions to this pattern.

When we consider the set of amino acids produced in Miller's revisited experiment [[13], underlined in Table 1], we find that the associations between Stickland pairs and complementary anticodons still hold, and the adapters form a cross-catalytic cycle (Figure 7). The significance of this observation is not about the plausibility of Miller's experiment as a model of the origins. What Miller-Urey synthesis suggests is that the amino acids are easily formed, with a yield that is somewhat inversely proportional to their chemical complexity. Overall, glycine and alanine are formed at a roughly 2:1 ratio, with a yield more than an order of magnitude higher than that of the rest of the amino acids [13], suggesting that alanine and glycine were the ancestral components of the genetic code, followed by valine and aspartic acid [50].

This initial amino acid composition is supported by the bias in the amino acid use of ribosomal proteins [51]. In other words, when the transition to proteins came, the simpler amino acids were used preferentially over the simpler ones; in particular glycine, alanine, and asparagine are overrepresented on the deep branches, although the case for valine, lysine and arginine remains unsolved (i.e. there seems that there was no significant increase in their usage, and that this has remained more or less constant) [51]. Here we find some evidence that supports the hypothesis; some of the anticodons for glycine (NCC) are complementary to some anticodons of alanine (NGC). This pair of amino acids is Stickland reactive (in fact amongst the most efficient pairs). A second step in the extension of the code is given by including valine and aspartic acid, which allow the associations gly-ala, asp-val and asp-ala, which are Stickland reactive, and their templates (NGC and NUC, respectively) form a small cross-catalytic system (Figure 6A-C). Thus the addition of two more adapters results not in two reactive pairs, but in three.

If an amino acid has the tendency to give a good energetic yield, it is expected to pair with several other amino acids of conjugate role. Strictly speaking, amino acids that tend to be good donors or good acceptors are expected to be assigned to anticodons that are flexible in pairing with other anticodons. For instance, it has been proposed that originally, glycine was encoded by the anticodon NCN, which would match the original anticodon of alanine, NGN. The latter additions valine and aspartic acid would have NAN and NUN respectively.

Considering an extension of the amino acids included in the code (at least as implied by the products of Miller-Urey synthesis) reinforces the correlation amongst complementary anticodons and reactive amino acid pairs. For example the addition of the next two amino acids, namely isoleucine and threonine (electron donors in the Stickland reaction), is accompanied by the implementation of new anticodons (NAU and NGU), which are still complementary to pre-existing anticodons of the electron acceptors (i.e. aspartic acid, NUN, and glycine, NCN; Figure 7). At this point, the "symmetry" of the primaeval code would be broken at the third position of the anticodons. However the anticodons for the electron acceptors (which are more numerous that donors), would be left intact. In this way, the multiplicity of the reactions would be maintained. Again, the number of possible reactions is increased, in a combinatorial manner.

**Figure 8:**
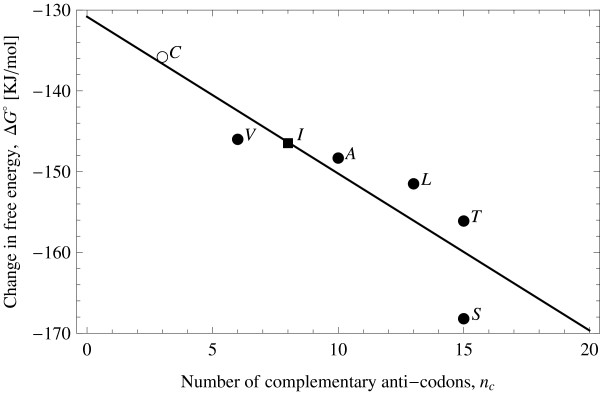
**Correlation between the "second redundancy" and the energetic yield of the Stickland reaction**. The line shows the linear regression (Δ*G*° = -130.823 - 1.94135*n*_*c*_; *r*^2 ^= 0.79; *p *= 0.017), computed using the available free energies for distinct electron donors when fermented against glycine (actual data indicated by circles) [37]. The square is a predicted value (not included in the fit). Filled symbols indicate Miller amino acids. A: alanine, C: cysteine, I: isoleucine, L: leucine, S: serine, T: threonine, V: valine.

Notice that there are two levels of redundancy that can support this multi-reactive proto-code. The first is to allow for synonymy of the anticodons associated with an amino acid: several anticodons coding for an amino acid will allow it to potentially associate with as many other amino acids as the degree of redundancy. This redundancy would eventually result in degenerated associations as in the extant genetic codes. The second redundancy adds to this by assigning the amino acids to anticodons rich in G+U. Each of these anticodons, through non-canonically pairing, are able to associate with several other anticodons, not just the cognate one. Examples of this secondary redundancy are the anticodons for serine and arginine, which can pair with up to 15 other anticodons in total, by both kinds of redundancy. More significantly, alanine's anticodons pair very well with anticodons of several electron acceptor amino acids: glycine, proline, histidine (whose codon would originally be assigned to aspartic acid), and arginine, making it readily available to react. This is because its anticodons are rich in U, which besides pairing with A, are able to pair with G. The other extreme is achieved by tryptophan and methionine, and have high A+C content, are not degenerate, and were unlikely to be present at the initial stages of the evolution of the adapters [50,51]. As a matter of fact, there is a significant correlation between number of complementary anticodons of an amino acid and the free energy of the Stickland reaction (Figure 8).

### Simulations

In order to explore the extent of this idea, a significance test was performed by resampling the genetic code and calculating the energetic yield of the Stickland reactions. First of all, amino acids were randomly assigned to an anticodon triplet to give a putative proto-code. Second, pairs of amino acids are allowed to react only if (a) they ended up assigned to complementary anticodons, and (b) they are Stickland pairs. These two constraints need to be considered explicitly in order to calculate the total yield of ATP. There are *n*_*r *_pairs of adapters that bear reactive amino acid pairs AA_*d *_and AA_*a*_, which react according to the equation

(1) $$\kappa_{d}^{\left(r\right)}\text{A}{\text{A}}_{d}+\kappa_{a}^{\left(r\right)}\text{A}{\text{A}}_{a}\;\overset{\text{ADP}}{\underset{}{\to }}\;\kappa_{p}^{\left(r\right)}\text{ATP + }\alpha \text{KA}{\text{c}}^{\left(r\right)}.$$

The stoichiometric coefficients *κ *depend on the specific reaction *r *[37]; *α*KAc^(*r*) ^denotes the *α*-keto acid produced in reaction *r*. It is assumed that there is enough ADP available to produce the necessary ATP. In order to compute the yield of ATP for the whole set of reactions, we first need to calculate the limiting reagent for each reaction. Then, considering the stoichiometry and that the reactions are completed, the ATP production can be obtained. First of all notice that the concentration of an amino acid that is involved in the reaction *r *is

(2) $${\left[\text{A}{\text{A}}_{i}\right]}^{\left(r\right)}=\frac{n_{r}}{\sum_{\rho \in R_{i}}n_{\rho }}{\left[\text{A}{\text{A}}_{i}\right]}^{sol},$$

where *R*_*i *_is the set of reactions involving the amino acid AA_*i*_, and [AA_*i*_]^*sol *^is the total concentration of the amino acid in the solution. Effectively, the concentration [AA_*i*_]^(*r*) ^is the concentration of adapters that have that amino acid AA_*i *_attached to it, and which are complementary to other adapters that have the Stickland partner attached. Because the stoichiometry, the limiting reagent of each reaction *r*, say, AA_*lr*_, will be the one with the lowest value of ${\left[\text{A}{\text{A}}_{i}\right]}^{\left(r\right)}/\kappa_{i}^{\left(r\right)}$. In that case, the yield of ATP for that reaction is

(3) $${\left[\text{ATP}\right]}^{\left(r\right)}=\frac{\kappa_{p}^{\left(r\right)}}{\kappa_{lr}^{\left(r\right)}}{\left[\text{A}{\text{A}}_{lr}\right]}^{\left(r\right)}.$$

The overall yield of ATP is simply the sum of the yield of each reaction. One mol of ATP gives roughly Δ*G*° = -80 KJ/mol.

**Figure 9:**
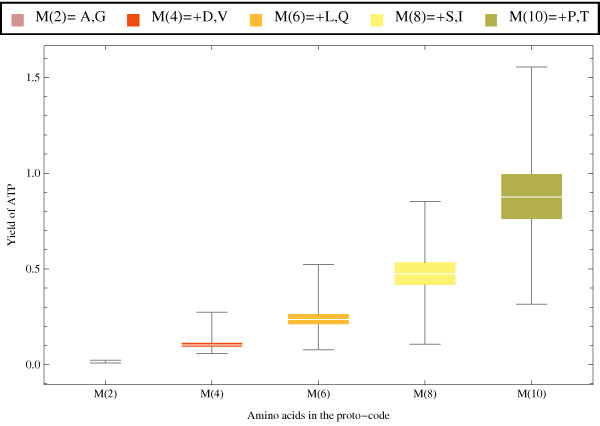
**Box plots of the distribution of ATP yield as a function of the number and composition of amino acids included on the code**. Distributions generated by bootstrapping the anticodon assignations 10^4 ^times under different compositions of *n *of the Miller amino acids, M(*n*), where *n *= 2, 4,6,8,10 (see legend on top). A = alanine, G = glycine, D = aspartic acid, V = valine, L = leucine, Q = glutamic acid, S = serine, I = isoleucine, P = proline, and T = threonine.

Code arrangements were bootstrapped 10^4 ^times, and the distribution of the yield of ATP was calculated under different subsets of 2,4,6,8 and 10 of the Miller amino acids (Figure 4). The mean yield of ATP, as well as its variance, significantly increases as the number of amino acids pairs included in the code increases (Figure 9). Therefore, under the assumption that the Stickland hypothesis is true, the inclusion of more amino acids could have been a driving force for the coevolution of the assignments. These distributions reflect the extent of the variability that can be available for selection to act. But in itself, these significance tests do not pose any statement about the evolutionary mechanisms that shaped the code. However, if there is any heritable mechanism capable of generating such variability in a proto-code, then the differences in the energetic yield can account for the relative selective advantage amongst types that employed different codes, or number or composition of amino acids.

Sequential bootstrapping was also performed in order to evaluate whether there is a preferential order of addition of amino acids. The different 18 combination of Stickland donors and acceptors of the Miller amino acids all lead to distributions of ATP yields with means that are different from zero (*p *< 10^-30^), which is to be expected. However, further additions of individual donors or acceptors, or pairs of them, resulted in a significant increase of the mean yield of ATP, whilst adding non-reactive amino acids significantly decreased the mean yield of ATP. This is reassuring, because it reflects the fact that the amino acids that can undergo fermentation, and can drive the expansion of the proto-code. But based only on the distribution of yield, this test does not allow us to derive any conclusions about the sequential increase of the amino acid repertoire.

In order to account for historical factors, a preliminary evaluation of the role of selection on variation was performed by implementing a Monte Carlo simulation. The central assumption is that there is a mutation mechanism that generates variants of the code, and a mechanism to select amongst these variants. For the mutations a swap-or-replace mechanism is assumed; two anticodon triplets are randomly selected, and if they bear different amino acids, the assignments are swapped. If they are the same, one of the amino acids is randomly substituted by any other present in the code. Selection acts according a Boltzmann factor, exp [-80(Δ[ATP])/*RT*], where the Δ[ATP] is the difference in ATP yield amongst the code that is implemented at any given time, and a mutated one; *R *is the gas constant and *T *the temperature. Then, "evolution" is allowed to proceed as a Metropolis algorithm.

**Figure 10:**
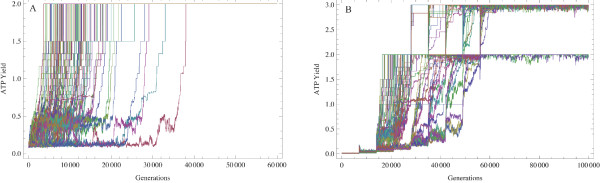
**Evolution of early codes by selecting on the ATP yield**. Evolution of the assignation of amino acids to adapters by selecting on the resulting ATP yield. (A) Employing only the simplest four Miller amino acids (alanine, aspartic acid, glycine and valine) 104 runs with initial random associations converged to optimise the ATP yield, in all cases reaching to the maximum of 2 moles of ATP. (B) 100 runs started from random associations to alanine and glycine and every 7000 generations a new Miller amino acid, randomly selected, was included and assigned to a random adapter. Most runs converged to optimal codes (with the maximum yield of 3 moles of ATP), but three did not.

One hundred and four replicas of the process were initiated with the following amino acids drawn with equal probability: alanine, glycine, aspartic acid and valine. All processes converged in between 5,000 and 30,000 generations (Figure 10A), and the resulting codes all produced 2 mol of ATP (the maximum possible according to the stoichiometry). The codes have an overrepresentation of the acceptor amino acids over the donors (Table 2). The latter, were almost invariably assigned to four of the eight codons composed exclusively of U+G, confirming the reasoning above (Table 2).

**Table 2:** Evolved proto-codes using the primaeval amino acids

Amino Acid	# anticodons (SE)	G+U content (SE)	*p*
Glycine	27.14 (2.8)	1.5 (0.80)	10^-3^
Alanine	1.96 (0.19)	2.98 (0.14)	10^-62^
Valine	1.93 (0.25)	2.96 (0.18)	10^-61^
Aspartic acid	32.96 (2.80)	1.33 (0.80)	10^-21^

Outcomes of selection on the fermentation yield of proto-codes randomly initiated with equal probabilities of glycine, alanine, valine and aspartic acid. For each of these amino acids (first column) the mean of the final number of anticodons that coding (second column) and the average of the G+U content of the assigned anticodons (third column). The fourth column is the *p*-value from a sign test centred at 1.5 (the expected mean number go G+U in a randomly constructed codon). The distribution of assignments are significantly different from 1.5. Pearson's *χ*^2 ^test on a binomial distribution was also applied, and in all cases resulted in *p*-values between 10^-15 ^and 10^295^. These test reject the null hypothesis, i.e. the structure of the proto-codes cannot be explained by random assignments. The data consists of the end points of 104 independent runs, each evolved for 60,000 generations (MC steps).

In another test, the code was allowed to sequentially include more amino acids; every 7000 generations each of the remaining Miller amino acids were assigned to a randomly chosen anticodon, until all of them were included into the code (Figure 10B). In 3 of the 100 runs some amino acids were lost and the codes converged to a sub-optimal state (with a yield of 2 moles of ATP). However, the remaining 97 converged to optimal codes, resulting in 3 moles of ATP (Figure 10B). Again, we find that the electron acceptor amino acids always are coded with high redundancy, and the donor amino acids with low redundancy, but assigned to anticodons with high U+G content (Table 3). In Table 4 some negative results are reported: the sub-optimal codes can all be explained by chance, suggesting that it is not only the composition of amino acids that matters, but also the assignment to proto-adapters.

**Table 3:** Evolved proto-codes, optimal solutions

Amino Acid	# anticodons (SE)	G+U content (SE)	p*	***p***^**+**^
Alanine	2.21(0.84)	1.98 (0.89)	10^-8^	10^-21^
Glycine	13.06 (2.96)	1.36 (0.66)	10^-7^	10^-9^
Aspartic Acid	14.24 (3.01)	1.25 (0.68)	10^-17^	10^-23^
Valine	2.19 (0.94)	2.23 (0.54)	10^-25^	10^-37^
Leucine	10.77 (4.60)	1.41 (0.61)	0.0024	10^-7^
Glutamic acid	2.09 (0.89)	2.18 (0.46)	10^-29^	10^-28^
Serine	2.13 (0.86)	2.15 (0.52)	10^-21^	10^-27^
Isoleucine	2.13 (1.12)	2.18 (0.54)	10^-22^	10^-30^
Proline	13.30 (3.04)	1.37 (0.69)	0.00003	10^-6^
Threonine	1.88 (1.16)	2.12 (0.56)	10^-18^	10^-21^

Outcomes of selection on the fermentation yield of proto-codes randomly initiated with equal probabilities of glycine and alanine. Every 7000 generations a new amino acid was added, randomly selected from the remaining Miller amino acids. Overall, the simulations ran for 100,000 generations (MC steps). The results are from 97 optimal codes (see Figure 10). * Significance p value for a sign test; ^+ ^Significance *p *value for a *χ*^2 ^test. Otherwise as in Table 2.

**Table 4:** Evolved proto-codes, sub-optimal solution

Amino Acid	# anticodons (SE)	G+U content (SE)	p*	**p**^**+**^
Alanine	4.67 (1.15)	1.93 (0.84)	0.18	0.12
Glycine	15.0 (3.61)	1.36 (0.69)	0.55	0.65
Aspartic Acid	3.67 (6.35)	1.00 (0.60)	0.23	0.24
Valine	2.33 (0.58)	2.00 (0.33)	0.13	0.14
Leucine	14.70 (1.15)	1.36 (0.56)	0.17	0.21
Glutamic acid	2.33 (0.577)	1.71 (1.57)	0.45	0.34
Serine	3.00 (1.00)	2.11 (0.61)	0.18	0.10
Isoleucine	2.33 (0.577)	2.29 (0.24)	0.02	0.03
Proline	11.30 (9.87)	1.35 (0.72)	0.39	0.77
Threonine	4.67 (4.62)	1.57 (1.03)	1.00	0.55

Outcomes of selection on the fermentation yield of proto-codes randomly initiated with equal probabilities of glycine and alanine. The results are from the 3 sub-optimal codes (see Figure 10). * Significance p value for a sign test; ^+ ^significance p value for a *χ*^2 ^test. Otherwise as in Tables 2 and 3.

Summarising, the principle of amino acid fermentation via adapter pairing can lead to particular structures of the code, and explain some features of redundancy. The results are statistically significant and reproducible.

### Possible experimental tests

There are two feasible experimental tests that can give support to the hypothesis above. First, notice that the "Stickland role" of any amino acid can depend on the actual partner. The classification shown in Table 1 is a compilation from the literature. In most of the experiments the amino acids were classified when tested against "universal" donors (usually alanine) or acceptors (usually glycine or proline). Ultimately, whether an amino acid acts as an electron donor or acceptor depends on its oxidation state relative to its partner, on their reactivity, and of the enzymes that catalyse the redox reactions. The electron-acceptor amino acids in all cases have higher oxidation states that their Stickland donors. This suggests that the roles of specific partners could be inverted, an observation supported by experimental results. For example, arginine can revert the roles of proline or lysine, and act as an electron acceptor when reacting with these [31,52], which is reasonable when we consider that arginine's oxidation state is higher than that of proline or lysine. Therefore, testing the reactivity and free energy of specific pairs of amino acids in the extant species that carry the Stickland reaction can convey more information about (a) fermentation pathways and reaction mechanisms that could have also been employed before the code was established, and (b) the energetic yield of these reactions.

The second possible test is related to the catalysis of the reactions. It is assumed that before the establishment of the genetic code ribozymes performed catalytic functions. The redox pathways, even when energetically favourable tend to be limited by very slow reactions and must be aided by cofactors and ribozymes. It would be possible to test some of the steps of the Stickland reaction, such as the deamination of the amino acids and the phosphorylation of the keto acids. Could ribozymes be evolved *in vitro *to catalyse these functions? This is a very interesting question to be explored in the future, for which there is an enigmatic starting point: glutamate dehydrogenase (which oxidises glutamate, as in the first step of the Stickland reaction) is an enzyme that has notable sequence homology to the synthetases [47,53]. This leads to the speculation that both functions could be somewhat related. It is a feasible idea to attempt to evolve the flexizymes (but in general, any other ribozyme) to have the dehydrogenase function.

## Implications of the hypothesis

### Structural constraints

Why would the proto-adapters form complementary RNA complexes? The attachment of the amino acids to the proto-adapters can allow their spatial coordination in such a way that ribozymes and/or cofactors can catalyse their coupled redox deamination.

If this coordination does not include a covalent bond to the RNAs, it is hard to rationalise any structural mechanisms, due to the immense possibilities of coordination for which we currently know no constraint. Furthermore a non-covalent mechanism could be limiting because of the slow speed of the reaction rates. But if the amino acids are covalently attached, then mechanisms for this reaction can evolve due to steric constraints.

Amino acids attached to an RNA complex (say, a double strand) could be more easily oriented and prone to be catalysed. Current tRNAs have their amino acids attached at the 3' end of the molecule, i.e., at position 76, whereas the encoding triplet in the stem is located between positions 70 and 72. In a double stranded RNA of 11 nucleotides the amino acids that are attached at the 3' end of the RNA do not lie close to each other.

**Figure 11:**
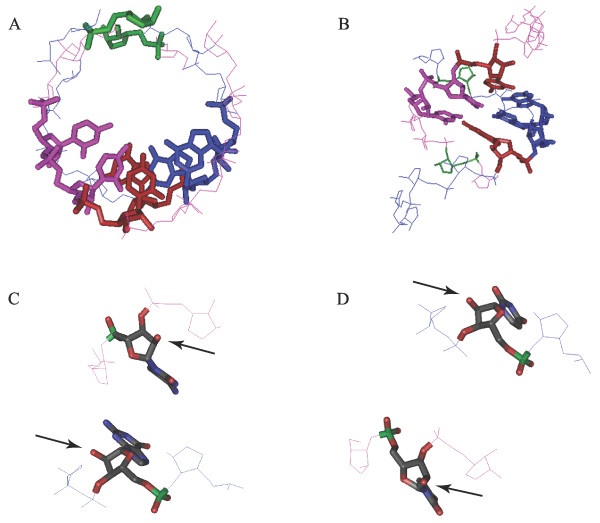
**Structural features of a double stranded RNA mini-helix of 11 nucleotides**. Structural features of a double stranded RNA mini-helix of 11 nucleotides. The backbones of each chain are shown in blue and pink wire representations. Their anticodons are shown in tubes; showing also the bases. The putative attachment points of the amino acids are shown in red (at the third base of the anticodon position) and green (three bases before the anticodon). (A) Top view. (B) Side view. (C,D) Close-up showing the spatial proximity of the attachment nucleotides (shown in tube representation, colour code according to atoms), and its neighbours (wire representation). The putative atoms of attachment (2'COH) of the ribose, indicated by arrows, project outside the molecule. The molecule was modeled from a known crystal structure [69, PDB ID:353D].

Because of the symmetry of the double helix, the attachment point of the amino acids to their RNA chains needs to be at equivalent positions. At the same time the amino acids need to have spatial proximity. The anti-parallel nature of the double helix gives two alternatives that satisfy these two constraints, and are shown in Figure 11A,B. The first possibility is that amino acids are attached to the third base of the the anticodon (highlighted in red). The second possibility is that the amino acids are attached three bases before the first nucleotide of the anticodon (shown in green). These are the only two options in an 11 nucleotides mini-helix where the amino acids lie in the same physical plane.

**Figure 12:**
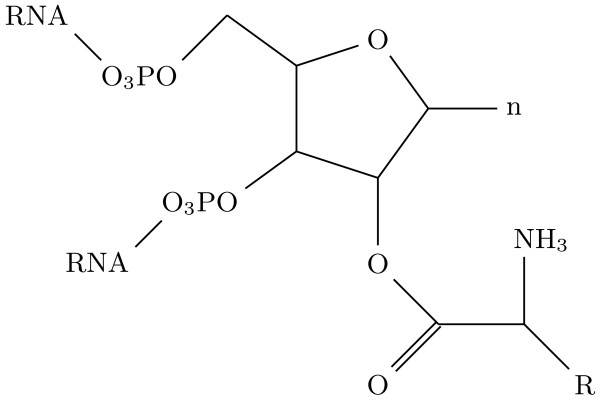
**Attachment of an amino acid to a ribose in an RNA**. One possible way of attaching the amino acid is a ester bond between its carboxyl group and the ribose's 2'C. R represents the side chain of the amino acid, and n the nucleoside.

In the tRNAs the carboxyl group of the amino acids form an ester bond with the 3' carbon of the ribose. However, at non-terminal nucleotides the 3' carbon is is occupied by the phosphodiester bond forming the backbone of the RNA. A sound alternative is to attach the amino acid to the 2' carbon of the ribose (Figure 12). The formation of the ester link has to be catalysed; in extant metabolisms, specific enzymes (the amino-acyl-tRNA-synthetases) catalyse the ester-bond formation. The flexizymes are artificially evolved ribozymes that have synthetase activity [54,55], thus this first step can be performed in the absence of proteins. Still, in order to form the covalent link with the ribose, the amino acid has to be activated (with AMP in the modern synthetase proteins as well as in the artificial ribozyme).

Figures 11C,D show a structural detail of the anchoring nucleotides facing each other, and arrows indicate the 2' point of attachment.

### Towards a reaction mechanism

The structural constraint posed above, although reasonable from the structural point of view, poses two problems. The first is that the deaminations are less likely to happen than for free amino acids. Current dehydrogenases do not form a covalent bond with the amino acid, for which oxidative deamination is more likely [28]. Thus if the Stickland reaction were mediated by ribozyme in an analogous way as the dehydrogenases, then the structural constraints described above would complicate matters. In fact, we can understand why. Because the ester bond displaces the electron cloud, the *α*-carbon is less electronegative; this impedes its deprotonation, making it unlikely that the nitrogen transiently forms a double bond with the *α*-carbon (the latter double bond is required for the nucleophilic attack of oxygen from water). Thus if covalently bonded, the amino acids are more stable than the keto acids [2]. However this does not take into account that the side chain of the partner amino acid can aid deprotonation by interacting with the nitrogen moiety. Naturally, there must be some cofactors (such as NAD^+^, or NADPH^+^) to mediate the electron transfer, and ribozymes that allow the reaction to proceed.

The following step is the reductive deamination of the other amino acid. This is a complicated reaction, and how the precise mechanism proceeds remains unclear even for the extant reductases [32-34]. The central limitation is that this deamination requires a very strong oxidising agent, which in the reductases involves intermediates between selenium esters and the deaminated glycine [32] (in principle an analogous bond could be established by thioesters).

The second complication is that after the deamination, the keto acids remain attached to the ribose. This is an important factor, because ATP can be synthesised only from the remaining keto acids, which need to be detached from the ribose. The ester bond between the carboxyl group and the ribose has to be broken (to result in a deoxyribose), by direct or indirect oxidative phosphorylation of the keto acid.

### Relation to the stereochemical hypothesis

The stereochemical theory suggests a physical and structural relation between the coding triplets (either codons or anticodons) and their cognate amino acids [[3], Chs. 6-7]. Based on sequence and structural data of riboswitches, it has been calculated that for some amino acids (arginine, histidine, isoleucine, leucine, phenylalanine, tryptophan and tyrosine) the chances that the coding triplets are evenly distributed in the binding site are small. The implication is that there is a significant association between the amino acids and some of their coding triplets (either codons or anticodons). Although only a fraction of the possible coding triplets (21%) show significant associations [5], these could have been an important determinant for coding, at least for some of the assignments [4,6].

Given that the Stickland pairing is correlated to anticodon pairing, in any random sample containing complementary anticodons the chances that Stickland pairs are formed are high. Thus the appearance of the two Stickland pairs in the stereochemical associations might just be casual. For instance, of the anticodons that are significantly associated with their amino acids in the riboswitch data, two complementary anticodon pairs appear: GUG (histidine) with AUA (leucine), and AUA with leucine (AUA) with GUA (tyrosine), although there is no data on whether these amino acid pairs are Stickland reactive. On the other hand, this would make the two theories compatible.

If both factors are taken into account, (Stickland pairing and stereochemical affinity), coding is more constrained; whilst the choice of some anticodons might have been determined stereochemically [6], their complementary pairs can be assigned to an amino acid that is an efficient Stickland partner. If this were true, it could explain why only a fraction of the amino acids are stereochemically assigned to their anticodons. Furthermore, it is also possible that the stereochemical factors bias the choice, purging the code of some naturally occurring amino acids that were not biochemically useful (for energy or otherwise). The analyses to support the stereochemical theory did not include the amino acids that seem to be most relevant for the initiation of the code, which are the simplest to synthesise abiotically, namely glycine, alanine, aspartic acid and valine, and other Miller amino acids. This does not discredit the stereochemical theory, but it does lead to some conflicts.

The stereochemical theory is an ideal precursor to the CCH hypothesis since it establishes some non-covalent associations that could become covalently linked in a later stage of evolution. In this way the non covalent associations would give birth to the catalytic cofactors. This view has its merits, but in the context of this article the sequence of events is somewhat reversed. Simple amino acids were used as energy sources (rather than for catalysis), and were initially assigned randomly. The more complex amino acids were included later in the code when biosynthetic pathways were available to synthesise them, most likely for catalysis. This is supported by phylogenetic analyses that showed that most of the species that have strong stereochemical associations to their coding triplets increased their representation much later in evolution, after the last universal common ancestor was established [51].

Other amino acids favoured in the stereochemical theory are underrepresented in the deep branches indicating that despite having good catalytic properties, they were hardly used in the nascent proteins. Thus, by the time these amino acids were included in the code, a more primitive code had to exist, and therefore the covalent associations to the proto-adapters (or even ancestral versions of the modern adapters) had to be readily established. Also, if it turns that the simple amino acids do not have a significant stereochemical association with their coding triplets, it is hard to argue for the case arginine and lysine, since they cannot be formed by simple abiotic processes, and thus cannot impose constraints in the early evolution of coding.

### Relation to the abiotic and early metabolisms

It has been argued that the citric-acid cycle might have originated in the opposite way [15,56,57]; instead of liberating CO_2_, it would employ it in a biosynthetic and reductive direction (as cyanobacteria and many extremophiles do), in accordance with the reductive atmosphere scenario [11,21]. This is similar to the scenario of the alkaline vents where CO_2 _is employed for the synthesis of the organic precursors. This theory is appealing not only because of it chemical basis [15,25], but also because of the geological context, which is know to exist [25,58]. It was mentioned in the introduction that it is sound to assume that the code originated around the time of the LUCA. If that is the case, were the early protobionts free from the geochemical substratum, or were they still strongly dependent on it? It makes a big difference for the following reasons:

• Under hydrothermal conditions (100°C) amino acid synthesis (not their breakdown, as proposed here) is energetically favourable, but at oceanic surface (18°C) breakdown is favourable [59,60].

• The melting temperature of the 11 nucleotide proto-adapter is between 30 and 50°C.

This means that under hydrothermal conditions the Stickland hypothesis does not work. What is worse, the whole theory of the concerted origin breaks down on energetic grounds, because proto-adapters smaller than about 60-70 nucleotides would not attain a stable fold (I am assuming the best case scenario where the proto-adapters are completely self-complementary, and therefore would have 30 to 35 paired bases, giving a melting temperature of about 100°C; interactions with ions and cofactors are being ignored). The situation is different away from the hydrothermal vents, where the double stranded proto-adapters are stable (the melting temperature is higher than the environmental), and amino acid fermentation is energetically favourable [59,60].

This allows us to hypothesise that there had to be a period where the autotrophic protobionts had to experience a cooler environment. This could have been a geologically cooler period, or perhaps autotrophs that gained independent compartmentalisation eventually migrated away from the mineral substrates at the vents towards cooler environments. In cooler environments the reaction rates are slower and any factor that increase catalysis, like the complex amino acids, are selectively advantageous, and thus proto-adapters could evolve to become CCHs. These organisms that were conveyed with the new catalytic innovations increased in frequency and spread. Upon confronting warmer environments again, an increase of the size of the adapters was necessary in order to allow thermal stability. Somewhere during those transitional stages, peptides, rather than cofactors, took over metabolic functions.

### Evolution of the genetic code

The code within the codon [61] reveals that codons GNN code for the four most abundant of the Miller amino acids, hinting at an ancient origin. As has been discussed above, these four molecules, if provided in abundant quantities, are enough to fuel metabolism. Although other Stickland partners can be found in the Miller amino acids, their negligible yield makes it unlikely that they could fully complement the bioenergetic requirements. But the code within the codon also reveals that in each biosynthetic pathway, amino acids derived from the primordial ones are assigned to codons that conserve their first position. For instance, codons starting with A code for amino acids synthesised from aspartic acid (asparagine, threonine, isoleucine, methionine, and lysine). Codons starting with C code for amino acids synthesised from glutamic acid (glutamine, proline and arginine). Codons starting with U code for amino acids synthesised by the Shikimate pathway (serine, phenylalanine, tryptophan and tyrosine). This strict pattern in the evolution of the code and of the amino acid repertoire was previously noticed and formulated as the coevolutionary theory of the genetic code [62,63], which states that precursor-product amino acids are assigned to codons that differ in only one base pair. The coevolution theory explicitly suggests the the distinct amino acids were not included in a random way in the code, but rather, that their biosynthesis evolved.

The assignment of the new amino acids to new anticodons had to be established through the proto-adapter-pairing mechanism described in the section "Evolution of the adapters", but with the slight difference that instead of attaching the same amino acid to an equivalent codon, it is the biosynthetically-derived amino acid that is attached to it. In fact, we do indeed find some signatures in the extant (universal) genetic code where the derived amino acids are assigned to similar anticodons: serine → glycine, glutamic acid → arginine, aspartic acid → asparagine, and threonine → isoleucine. In addition, we also find threonine → methionine, which are "brothers" (both derived from aspartic acid) rather than precursor-product, but since they share intermediates, it is plausible that there were pathways that could convert one to the other. Table 5 summarises the pathways involved in these assignments.

**Table 5:** Assignation of biosynthetically derived amino acids to new anticodons

Precursor amino acid	Original anticodon	Intermediate complement	New assigned anticodon	Derived amino acid
	GCC	GGC	GCU	
Glycine	GCC	GGU	AUC	Serine
	ACC	GGU	ACU	

Aspartic acid	AUC	GAU	AUU	Asparagine
	GUC	AAC	GUU	

Glutamic acid	CUC	GGG	UUC	Arginine
	UUC	GGA	UCU	

	AGU	AUU	GAU	
Threonine	AGU	AUU	AAU	Isoleucine
	GGU	AUC	GAU	

This begs the question of whether the biosynthetic modifications improve the fermentation yield. By changing the oxidative potential in the biosynthesis, the Stickland role can be altered, as in the case of the interconversion of serine and glycine, or aspartic acid and asparagine. In the other cases the Stickland role is conserved. At the moment the evidence is not conclusive, although by considering posterior modifications to the code we might gain considerable insight. Nevertheless the biosynthesis of amino acids could have evolved after selective pressure was established by the use of amino acids that were scarce but beneficial, and which were therefore already assigned to some anticodons.

### Concluding remarks

The Oparin-Haldane hypothesis, and Miller's experiment were major contributions to the understanding of the material basis of life. However, after the discovery of the genetic code, it became obvious that it is much simpler to synthesise the simple building blocks of life, than to assemble them in units that are capable of evolution. The RNA world has become one of the central paradigms for this early stage of life, but how it emerged remains unexplained. The origin of genetic code is one of the most puzzling questions about the transition from this RNA world to the modern modes of life. The pervasiveness of the genetic code in the tree of life, with its minor deviations, suggests that it must date back to the last universal common ancestor.

The hypothesis presented in this article complements the current understanding stating that the amino acids and proto-adapters were added to the code in pairs [6,44,45]. Stickland pairing is an appealing candidate mechanism because (a) it imposes constraints to the amino acids to be assigned to newly evolved proto-adapter pairs, and (b) it favours redundancy by assigning good electron donors or acceptors to proto-adapters in a way that allows it to react with several amino acids with complementary Stickland role. The evidence that supports or that is in favour of the hypothesis can be summarised in the following points:

• Bacteria of the genus *Clostridium *employ pairs of amino acids as energy sources.

• The energetic yield of amino acid fermentation is comparable to that of carbohydrate fermentation [36].

• In the abiotic syntheses of amino acids, Stickland pairs are readily produced.

• The composition of the ribosomal proteins of early organisms is biased towards the use of the simpler amino acids, specially alanine, asparagine, and glycine [51].

• The code within the codon suggests the ancient origin of the four basic amino acids alanine, aspartic acid, glycine and valine [61].

• Amino acids that are favoured in the stereochemical theory [5] are underrepresented in the deep ancestral branches [51], implying the coding might have been established earlier by other means.

• Several anticodons that are complementary are assigned to amino acids that are conjugated Stickland pairs, which include the simpler Miller amino acids (Figures 6 and 7).

• A significant correlation exists between the number of complementary anticodons of an amino acid and the free energy of the Stickland reaction (Figure 8).

• The melting temperature of the 11-nucleotides proto-adapter is compatible with the conditions at which amino acid degradation is thermodynamically favourable (30 - 50°C) [59,60].

• By increasing the number of amino acids used as substrate, the numbers of possible reactions is increased in a combinatorial manner.

• The multiple complementarity of the proto-adapters (by considering U-G pairs) increases the synonymy of the reactions.

• The inclusion of more amino acids to a proto-code can increases the ATP yield, especially if refined by selection (Figures 9 and 10).

As mentioned above, we need to invoke a specific evolutionary model in order to account for plausible evolutionary histories. This in itself is an independent research subject; for example, we might make different assumptions about the modes of selection: did it act in a directional way, or was it stabilising? What kinds of costs could have been involved in the inclusion of new amino acids to the early metabolism? To what extent were the codes heritable? What is the distribution of mutation effects on the adapters? Questions such as these are relevant in determining the details of the associations. Fortunately, these are accessible by combining RNA models with state of the art of evolutionary genetics theory.

But even sparing from these details, the theory proposed here helps us to understand the early steps in the establishment of the code. It explains stages prior to the CCH in terms of bioenergetic advantages to a hypothetical protobiont living prior to proteins being implemented for enzymatic and structural roles. Although we can not know with certainty what actually happened, the ideas exposed in this article give a testable coherent picture about some factors that could have moulded the genetic code.

## Competing interests

The author declares that he has no competing interests.

## Authors' contributions

HPdV carried out all analyses and simulations and wrote the text.

Reviewer's report

**Title: **Bioenergetic origin of the genetic code

**Version: **2 Date: 27 June 2011

**Reviewer: **William Martin

**Reviewer number: **1

Report form:

Review of Vladar for BD

*The author suggests that there is a connection between the structure and evolution of the genetic code with the Stickland reaction, a fermentation reaction in anaerobes like Clostridia and relatives*.

I would like to start by clarifying that I do not claim that the extant pathways of the Stickland reaction are directly derived from the putative ancient pathways for amino acid fermentation, as discussed in the main text. But the existence of these pathways in extant organisms make amino acid fermentation plausible.

*"The proposition is that the origin of the genetic code traces back to the origin of metabolism", that is a nice thought but Copley et al. (2005) should developed that previously, but from a very different angle*.

The mechanisms presented by Copley et al (2005) [2] predict that several of the amino acids can be synthesised from their corresponding keto acids in dinucleotide complexes. The dinucleotides match the first two codons of the amino acids in the code; this provides a possible mechanism for the coevolution theory of the code [62], and to the code within the codon [61]. Personally, I regard the biosynthesis of amino acids as a later stage in the evolution of the code. This is particularly appealing from Copley et al's (2005) [2] ideas; the amino acids, synthesised in dinucleotides corresponding to codons, would be transferred to adapters in a synthetase-like reaction. This would necessarily force codon swapping and reorganisation of an existing proto-code. But at this point the function of the amino acids would have departed from bioenergetics, otherwise we would have a circular argument and a *perpetuum mobile*.

*On the topic of amino acids and bioenergetics, Amend & Shock (1998) *[59]*show that the synthesis of amino acids (and proteins) from H*_*2 *_*and CO*_*2 *_*and ammonium is thermodynamically favourable under hydrothermal vent conditions. Amend and McCollom (2009) *[60]*show that the synthesis of cell material from H*_*2 *_*and CO*_*2 *_*and ammonium is thermodynamically favourable under specifically alkaline hydrothermal vent conditions, whereby the synthesis of amino acids (not their breakdown via the Stickland reaction) delivers the strongest contribution to the overall exergonic reaction*.

The conditions in which amino acid fermentation is exergonic happen to be the same as those maintaining the stability of an 11-nucleotide proto-adapter. However, under the conditions of the hydrothermal vents, where amino acid fermentation is not favourable, the proto-adapters need to be much larger to allow for thermal stability of their structure. In the text I suggest that the initial steps for the establishment of the code could have occurred in cooler periods (quiet or intermittent episodes of vent flux), or away from the vents location. This would favour both amino acid breakdown and proto-adapter stability.

*More generally, the notion that these amino acid pairs might somehow interact in such a way as to ultimately lead to the synthesis of acyl phosphates when they are connected to tRNA (see *Figure 12*) is not going to work at all, because the carboxyl group is esterified in RNA-bound amino acids and that ester bond formation requires ATP hydrolysis at least in modern metabolism ("activation" of the amino acid) so there is no room for net energy conservation*.

The chemical details of the RNA double strands to which amino acids are attached is only one of several possible interpretations, but one for which we can rationalise certain details. There is no evidence that stereochemistry can explain the association between the simpler amino acids and RNAs. Thus I appeal to chemical bonds catalysed by ribozymes (synthetases), which do have sequence specificity. This structural model is perhaps the most na"ıve interpretation of the correlation amongst the Stickland complementation and the anticodon complementation. Accurate structural analyses, both computational and experimental, could establish in more detail the nature of the interactions between amino acids and RNAs. But in any case, energy has to be invested in order to establish a covalent bond. In fact, as you point out, the synthetases, in both versions extant proteins and ribozymes (flexizyme), need amino acids that are activated with AMP in order to be transferred to the adapters. In an iron-sulfur world, the activation could be achieved by thioesters, but this does not avoid the energetic investment.

*What is the nature of the chemical bond between the AAs and the RNA in *Figure 2B, *a 2' ester?*

This is an appealing possibility, considering that the synthetases (natural proteins and selected ribozymes) form this type of bond. First of all, position 3' in my molecular model (Figure 12) is occupied by the phosphoester of the backbone of the RNA molecule. Thus the only free reactive group is the -OH on the 2'carbon. The activated amino acid can form this bond because the oxygen in position 2' makes a nucleophilic attack to the beta-carbon of the activated amino acid (the one linked to the phosphoester). Without the AMP, the nucleophilic attack would not proceed. Furthermore the synthetases of class II (ancestral mode) amino-acylate the carbon 3', not the 2' as in my mechanism. But on the other hand, some flexyzimes charge the adapters at carbon 2' when targeting a non-terminal nucleotide. Therefore it is not inconceivable that synthetases originally acted in this way.

*The concept as suggested in the title is problematic because there have to be very large amounts of amino acids as an energy source running through this system if the Stickland reaction is really going to serve as a bioenergetic motor, having simpler compounds like H*_*2 *_*and CO*_*2 *_*as the energy source at the origin of the genetic code with amino acids doing things like catalysis and making proteins might seem more reasonable*.

In fact, I agree that simpler compounds could be used as energy source. My point does not need to be regarded as contradicting this. Instead, what I claim is that using amino acids as an energy source is plausible on energetic grounds, and that this links to the early stages of assignment to proto-adapters. This does not imply that amino acids are the main energy source, or that they need to replace other sources, etc. The central point, as I explain it in more detail in the new version of the manuscript, is that prior to the usage of amino acids as catalysts, the assignment of the simpler amino acids (most of which are poor catalysts) to complementary proto-adapters can account for the earliest steps of coding.

*The chemical connection -- at the level of structure that we can draw -- between amino acid pairs and short RNA intermediates en route to the code is much much weaker (if at all existent) than the logical connection that can be construed as in *Figure 2*or *Figure 11.

We are dealing with factors that occurred long time ago, and only rarely we can have data to draw hypotheses about their origin and evolution. It is even more unusual to be able to test these ideas. My hypothesis is: (a) logically sound, (b) supported by (preliminary) evidence, (c) chemically plausible (in the sense that amino acid fermentation can sustain metabolism). The evidence is weak, but sufficient to draw some preliminary conclusions which merit further research. I accept your criticism based on chemical grounds that this structural model is unlikely, and thus a "weak connection". It does not help that we have no understanding about the ribozymatic machinery that was available at that time. In any case, these structural details are neither the centre of the hypothesis, nor ultimately relevant for evolution. Of course, selection has to act on a material basis, which might impose important constraints. Therefore, if the hypothesis presented here can explain some early steps of the code, the "weak chemical connections" will reveal the key aspects that we ignore about the ribozyme metabolism.

Reviewer's report

**Title: **Bioenergetic origin of the genetic code

**Version: **2 Date: 4 August 2011

**Reviewer: **Eörs Szathmáry

**Reviewer number: **2

Report form:

*This is an extremely original idea that is a pleasure to read. For quite some time I have been wondering what the initial advantage of amino acid usage could have been. Every such advantage is suggestive of a possible preadaptation (exaptation) that has the potential to render further evolution easier. Let me discuss some issues in steps. First, note that in an RNA world some amino acids may have been present already because they played a role in nucleotide and coenzymes biosynthesis (Gly, Ala, Val and Asp are the prime suspects)*.

These "Miller" amino acids are the simplest to synthesise by several means. Furthermore, the phylogenetic signal in ribosomes strongly suggest that these (amongst other small set) were preferentially used during the early days of the translational machinery. The implication is that (be it by historical contingencies, or by selective fixation) these amino acids were fundamental to the RNA world metabolism.

*As Koonin suggested, selective retention of such amino acids by nucleic acid moieties to prevent them from passing through an early, leaky membrane was potentially selectively advantageous*.

The selective retention is an appealing mechanism, in particular when we consider that as far as we know, there are no ribozymes associated with permeability and transport. Thus alternative processes like the one you mention, had to exist in order to deal with the loss by diffusion through a membrane. This might have been a crucial step, in that the association between amino acids and oligonucleotides was not necessarily specific. This would have created an initial diversity within which further selective processes (e.g. fermentation, or any other) could act.

*The catalytic role, as suggested by the CCH hypothesis, would have to come later. I see the bioenergetic hypothesis as an attempt to build an even stronger bridge between very modest usage of amino acids and their usage as catalysts*.

Certainly catalysis is the central function of amino acids, and as yourself and Kun have previously showed (2007), aspartic acid is very reactive. The problem, as discussed in the text, is that the stereochemistry has not been shown to be a determinant of the coding triplets for the simpler amino acids. The bioenergetic role of the amino acids accounts for some assignment patterns after selection has acted.

*De Vladar proposes a further pattern for the vocabulary extension of amino acids, in that he points out that in several cases amino acids assigned to complementary anticodons are Stickland pairs. Well spotted! Incidentally, it is also true that they tend to be complementary in the catalytic-structural role, as the Rodins and I noticed before. The bioenergetics idea is so nice that I hope that there is something in it, but careful further thinking is badly needed (not necessarily in this pioneering article)*.

I regard complementarity of amino acid roles as imposing strong constraints on the establishment of the code. Whether explicable through the Stickland reaction or through a catalysis/structural function, it is still a question of developing detailed arguments and gathering further evidence. For example, we could regard the whole complex of two proto-adapters with amino acids attached to them as a more complicated version of the CCH, which allows a combinatorial range of ribozymatic functions even considering only the simpler amino acids. The appeal of the subject is that we can test these kinds of hypotheses!

*A brief technical note. Although originally the coding coenzyme handles were proposed to be anticodon triplets, the modern version proposes that the advent of CCH arrived with short loops. There are two arguments to support this. First, specific recognition through Watson-Crick pairing, and ample residence time on the ribozyme to be catalytically complemented, the handles must have a fairly defined conformation, which is ensured by loops but not free triplets. Second, the tRNA evolution consideration with the Rodins also point to the appearance of anticodon and catalytic amino acids at the short loop stage*.

These two arguments also apply to the Stickland hypothesis. Nucleotide triplets have a melting temperature that is too low for dimers to form in solution. However, proto-adapters of a dozen of nucleotides can be stable at physiological (although not at hydrothermal-vent) temperature. Thus it is most likely that the three dimensional conformation of the RNAs play a crucial role in the function, and consequently on the eventual establishment of the code.

My first worry is the whole context of the bioenergetic role. As the author cites, there are chemical transformations (oxidation and reduction) that happen here. Does he think that these are spontaneous, once aligned by the complementary handles, or is further catalysis needed? If yes, why not simply assumes ribozymes to bind the two reactants?

Initially, I had the hope that the structural constraints on the handles would facilitate proton and electron exchange. But a closer look revealed that the reactions are less prone to happen when covalently bonded. On the one hand this allows, as you say, ribozymes to act on a very specific moiety, but on the other hand, it suggests the need to employ strong reductant cofactors. So I presume that both cofactors (such as NADH or equivalent) and ribozymes are needed. This is my assumption.

*Furthermore, what happens to the transformed reactants afterwards? If they remain linked to the same handles, then each handle would ultimately be linked to a diverse set of different intermediates of metabolism*.

This is a question that depends in a very specific manner on the actual mechanism. As I can imagine it now, is that after the deamination, there is an elimination of the remaining moiety (the keto acid) by removing if from its carbonyl group and synthesising, for example, acylphosphates. The problem here is that this requires a reductase activity -which certainly is not spontaneous- and needs a strong reductant cofactor (extant Clostridia employ seleno-protein complexes). Although problematic, this is a critical step, since the deamination itself, although transferring electrons from one amino acid to another, does not release free energy.

*This leads to my second worry. How does assignment (coding) arise? What is its significance? The bioenergetic role by itself would not call for coding. Does de Vladar think that assignment just happens, through stereochemistry, and gets frozen in the system for a while, without any functional role? Note that for example in the CCH hypothesis coding naturally arises through the necessity to bind the right amino acids through their handles, to catalyse the right reactions by the complemented ribozymes*.

The bioenergetic role imposes constraints on the pair of adapters, but it is true that it does not itself prescribe any specific triplet to any amino acids. However, if one triplet is set, the choice of the amino acids that can be assigned to the complementary anticodon is cut by half. For example if there were only four amino acids in question at the initial stages (ala, gly, asp and val) then the choice of assigning a correct (Stickland complement) amino acid is only of one in two. However the complements of the complement via U/G pairs in the second position would constrain the amino acid which could be assigned to that codon (the one having the same Stickland role as the original amino acid). Thus the degrees of freedom are constrained. However, as shown in the text, initial random assignments of a few amino acids (2-4) could be enough to harvest limited energy. Then selecting on this energy yield results in more specific patterns. However, at some point we will need to invoke the synthetases. These are the responsible for reading the sequence of the proto-adapters and charge them accordingly. In the simulations, all assignments were equally likely, and neutral. As it has been shown by yourself and the Rodins [6], this is not the case; the synthetases arose as a need to make specific (or pseudo-specific) assignments. Some insights come from the crystal structure of the flexizyme, showing that the amino acylation occurs by stacking the phenylalanine ring with a guanidine ring; the former is stabilised by the oxygen of the latter. This orients the carboxyl with the 3' carbon of the terminal adenine, so that the bond can be established. Therefore stereochemistry (although somewhat different than the stereochemical theory) shows that the assignments can be substantially biased.

*Third, the nature of the reactions with the amino acids bound. Assume that Figures *6 and 7*actually do present pretty well what is imagined to have happened. There are a few difficulties. As I noted above, mere triplets may not be sufficient for such an interaction because of their weak binding to each other and also to the amino acids (selected Yarus aptamers are always bigger)*.

I do not assume that these are triplets; Figures 6 and 7 show only the triplets in order to sketch the cycles that can be formed due to the complementarity of the proto-anticodons! I have made this more explicit in the new version.

Furthermore, I am worried whether the two amino acids at opposite ends of the complementary strands would be free to interact or not. Perhaps not!

This is an excellent observation. In short, if the amino acids are attached at the end of the chains they are not close enough to interact (Figure 11). Thus I had to figure out at which positions this could happen, shown in Figure 11. Notably, the ribo-synthetases can amino acylate at arbitrary positions of the RNA, not only at the end of it.

*Setting this aside, what is the chemical nature of binding of the amino acids to the handles? I suppose De Vladar thinks (with me also) that the link was covalent at this stage. But how? Something very important may be lurking here. For the CCH hypothesis I was prompted to assume that the initial coupling must have been a stable N-link as seen in some contemporary amino acid-based modification to the anticodon loop. Later I realised that I have simply forgotten that Woese suggested the same in the late sixties already. In a letter to me he wrote that "now I would be worried about the energetics of this reaction". Yes, yes, but here may just be a crucial link! Let us think about it in the future. I hope readers do not mind that I am thinking in writing here*.

The advantage of assuming an N-link is that the reactions could be related to the synthesis of nucleotides. The problem is that if linked through the nitrogen, the reactions that can happen afterwards do not change the oxidation state of the molecule; but this debatable since it would all depend on the specific cofactors and ribozymes. But a priori, the odds favour a more labile ester link, a conformation in which the oxidation-reduction reactions can happen.

Reviewer's report

**Title: **Amino acid fermentation at the origin of the genetic code

**Versions: **2 & 3 Date: 22 August 2011/12 December 2011

**Reviewer: **Ádam Kun

**Reviewer number: **3

Report form:

*Harold P. de Vladar presents an intriguing hypothesis about the origin of the genetic code. He finds correlation between the code and reactive pairs of amino acids that could be used to fuel a metabolism, as seen in extant bacteria in the genus *Clostridium.

*I fully agree with the statement that "we cannot possibly know what actually happened", thus there is a need to come up with plausible and testable hypotheses about the origin of life and its stages. However, I think, that amino acids were first and foremost catalytic help, and not a source of energy*.

I am in total accordance with the idea that when the translation machinery was about to be established, the amino acids had foremost a catalytic (and structural) role. Critically, it is precisely this function that must have triggered the evolution of the translation machinery. Most likely, but still debatably, this function had to be implemented even before such machinery existed. Needless to say, this does not impose any constraint on the history of the role of amino acids in an ancient metabolism. Although amino acids are relatively simple, they are reactive, have a high oxidation state, and are easy to synthesise. It is therefore to be expected that they serve(d) many purposes. The ideas that I have presented, as discussed, are not inconsistent with the usage of amino acids as catalysts. In the metabolism of extant organisms, amino acids are not used only for catalysis or structural functions in the proteins; they have a variety of functions. Any combination of these (or other) functions might also have been present in earlier stages of evolution. In my opinion, we are lucky that we can find ways to rationalise any of these functions. The ideas presented here might be wrong, but at this stage, the relevant issue is that we have information enough as to state a precise hypothesis and devise ways to test it.

*I base my assessment on the following lines of argument: (1) Historical contingencies: We find "fossils" of the past in our current metabolism. The RNA centric translation with a ribozyme doing the peptidyl-transfer is a fossil from the RNA world. Our coenzymes, which all harbour a nucleotide part, even though it is not the nucleotide part that does the job, are again fossils from the RNA world. The chemical nature of the bases is a contingency, there are many possible alternatives, some might even be better than these, but once evolution found this solution, it is very difficult if not impossible to change them. If amino acids had had such an important, central role in energy metabolism it would show in our current metabolism. The fact that there is an example of this in one genus of bacteria, but nowhere else in the living world does not help. If Clostridium would be the most ancient bacteria, so that this mode of energy metabolism is reserved here, but not in the other lineages, then it would be a valid argument. However, it is not the case (correct me if I'm wrong)*.

I agree that the idea is puzzling, and that the evidence is not the as strong as it might be. Yet, how much about the bacterial biota do we know? As you say, "once evolution found this solution, it is very difficult if not impossible to change them". But is it not exactly because of this tendency that you point out that it is in the differences that we often find crucial clues? After all, if glucose fermentation was the optimal solution, why are there other types of fermentation? Almost literally, every new bacterial species (in the broad sense) whose metabolism is surveyed unveils a new dimension of pathways. Until recently, there was a strong bias towards detecting microorganisms that use carbohydrates as an energy source. The odds are in favour of detecting alternate pathways when we discover new species. Thus I appeal to the factual cliché that absence of evidence is no evidence of absence. But at the same time, I would not wish to bury my arguments in obscurity until these are explored. I accept the criticism exposed above, particularly as a further opportunity to add or remove support to the hypothesis. Doubtlessly, once the diversity of metabolism is less biased and we have clearer ideas about the raft space of possibilities, we will be able not only to evaluate this hypothesis more accurately, but to state many others as well.

As a more direct answer to your concerns, although it is true that most known microorganisms do not preform the Stickland reaction, the proteins employed for this pathway are widespread. In particular, the reductases and the dehydrogenases perform the critical steps in the amino acid fermentation. These enzymes are not exclusive for the Stickland pathway, although in *Clostridia *they seem to be specialised for that function. The fact that these reactions happen allows for the possibility that analogous mechanisms existed, a possibility that was proposed at two points in the text: first, when it was suggested that the prebiotic mechanisms could be analogous to those performed by the enzymes above. Second, when it was proposed that RNAs can be artificially evolved to perform such functions, for which we would need to provide some cofactors (electron carriers, most likely NADH), as we learned from the biochemistry of the amino acid fermenters. Naturally, a detailed analysis of the molecular mechanisms may reveal molecular fossils pointing to factual and specific evidence.

*Furthermore, in an RNA world setting we can safely assume that there are ample sugars around (if nothing else, the nucleotides can hydrolyse to give ribose), which are much better energy sources than amino acids (see 3rd paragraph of the "The Stickland reaction" section)*.

If sugars were vastly available, they could have a major energy source. If they were limiting, then a "division of labour" would be convenient, with sugars employed to synthesise nucleotides and amino acid fermentation to fuel metabolism. Incidentally, nucleoside biosynthesis might just as easily be synthesised from glycine and aspartic acid [15]. Therefore both sugars and amino acids are needed for the synthesis of RNAs. In any case, the view of a "main energy source" might be biased, and inapplicable in a prebiotic scenario, because most compounds were scarce (which is what most prebiotic models suggest, particularly away from the hydrothermal vents). Thus harvesting energy from multiple sources would be a convenient bet-hedging strategy. The existence various carbon sources does not contradict the amino acid fermentation arguments for the origin of the code.

*(2) Prebiotic synthesis of amino acids: Most of the amino acids in the genetic code have a rather low yield in the Miller experiment. I agree that the possibility of their formation is the most important outcome of the experiment, and there can be other, prebiotically plausible reaction pathways that produce amino-acids in much higher yield. But as de Vladar states "But was the relatively low abiotic yield of amino acids enough to sustain protobionts based on RNA metabolisms? The answer to that question strongly depends on the role of amino acids at the moment when the genetic code was established." (last line in the section "Abiogenesis of amino acids") Indeed, if amino acids are used to fuel metabolism, then they are consumed in the process, thus requiring a much higher yield than using them as cofactors, in which case the amino acids are not consumed*.

Admittedly, the question of the yield of amino acids still stands. This is a question about geochemistry, not about evolution; it is nevertheless relevant. As I see it, what is most important about Miller-Urey synthesis, is that it shows the ease with which different amino acids are produced. The yield in this particular experiment is somewhat moot, because the conditions under which amino acids were formed are largely unknown. What is clear is that amino acids are conspicuous products in organic chemistry, and that the simplest ones (glycine, alanine, etc.) are the most common. Recall that under a range of conditions [12,64] and energy sources [11], and as well as from cosmogenic synthesis [65], these and many others amino acids are formed. Moreover, Bada [66] calculated that the amount of amino acids in non-biotic reservoirs is larger than in the biosphere. Thus it is specious to say that abiotic amino acids sources were not available.

However, these patterns cannot be explained by catalysis alone, because besides aspartic acid, the other simple amino acids are bad catalysts. Hence it is doubtful that a specialised ribozymatic machinery (synthetases) would had evolved in order to charge RNAs with the simple, non-catalyst amino acids; there would simply be no selective advantage for such a system, and instead only costs.

Thus while the first part of the statement "The synthesis of most catalytically important amino acids is very elaborate, and their abiotic yield is negligible (except for aspartic acid)," is true, it does not imply the second part: "a fact that necessarily postpones the catalytic functions of the amino acids to later historical stages."

Indeed, aspartic acid could have functioned as a catalyst from a very early age. My statement referred to the full repertoire of amino acids; in order to function as a robust battery of catalysts, would have to (and continue to) do so only at later stages once their biosynthesis had evolved (this is independent of whether there was a fermentation role or not for the simpler amino acids at earlier stages). The central argument is that Stickland type reactions can explain some patterns in the code. A collateral advantage could come from catalysis, setting up the pre-adaptations for a catalytic rolls (as in the CCH).

*(3) Development of the RNA world: I have reservations about the historical context of the presented hypothesis. The current view of the RNA world puts the evolution of the genetic code at the end of the era*.

I completely agree. The ideas that I present in the article intend to explain the first steps, the pre-adaptations, that eventually led to a code. The rearrangements (fine-tuning, if you wish) of the code to optimise catalysis, protein folds, etc. would come towards the end of the RNA era, as you point out. But the earlier steps might have occurred much long before that, say, after the iron-sulfur era, at the hydrothermal-vents.

*The RNA world has already possessed cellular organisation and a rich metabolism run by many ribozymes before the advent of translation. Thus an energy producing system was already in place. In view of our current metabolism, and what was surely available to the RNA world, sugars and simpler organic compounds were the main sources of energy*.

I restate: using amino acids as energy sources is not incompatible with their use as catalytic factors and thus also not with the evolution of translation. But there is little doubt that saccharides were the main source of energy at that point, in part because they are essential for (a) nucleic acid metabolism, and (b) membrane regulation in the absence of proteins, and it is therefore hard to exclude saccharides at that stage. But good as they are, the saccharides (particularly the hexoses) are too reactive, and need to be under strict control due to the risk of glycation of both lipids and amino acids (although reactions with the later might have had a role in RNA synthesis). Thus if we focus on earlier stages, less risky, but equally efficient carbon sources would be amino acids, other small organic molecules, and of course CO_2_.

*An energy metabolism based on pairs of amino acids attached to specific adapters would require 10 (limiting the amino acid set to the 10 primordially available in *Table 1*) highly specific enzymes to ligate the amino acids to the adaptor. Such highly specific system could only arise at a stage with an established metabolism*.

I avoided going into this subject on the article on purpose, since it is a complex subject, but it is a very good observation. The enzymes that attach the amino acids to the tRNAs, namely the synthetases, are highly specific to both of their substrates: the amino acid and their cognate tRNAs. Their evolution is in itself puzzling [67,68], but this specificity is what is thought to have shaped the code [6,67,68]. But ribozymes have been artificially evolved to perform the amino acylation reaction [54,55], which is a rather encouraging piece of evidence. To summarize, the picture is that the evolution of the synthetases was boosted by the emergence of new modes of amino acylation, which also allowed the inclusion of new amino acids to the code. But this had to happen with substantial variability in the proto-adapter sequences. It had to be a very specific metabolism, but probably ribozymes and cofactors were enough for these early steps, as suggested by in vitro evolution of ribo-synthetases [54,55].

*However, such an established metabolism does not generally need new energy sources*.

I think that from the perspective of the ribozymes, the energy source does not matter. In fact, if the energy metabolism is canalised into a single currency (ATP), the rest of the metabolic system is blind to it, regardless of what the source is, without needing major rearrangements when new energetic sources are implemented.

Is the Stickland reaction that much efficient compared to other modes of anaerobic fermentations?

The oxidation states of the amino acids are similar to those of carbohydrates, and if efficiently reduced, they can synthesise a similar amount of ATP. For example, the fermentation of glucose leads to 102 KJ/mol ATP, lactate to 71 KJ/mol ATP, and the fermentation of two glycine molecules results in 72 KJ/mol ATP.

If amino acids were so abundant, that they would be a convenient source of energy, then why amino acids that are very abundant in the Miller experiment are not represented in the genetic code (e.g. sarcosine, N-metylalanine, etc., Miller and Urey 1959 Science 130:245)?

This concern is a central question, and perhaps the catalytic idea along with the mechanisms for modifications to the code, can be a better avenue to explain the amino acid repertoire. But it is important to consider that the Stickland reaction happens with amino acids that are not the proteic ones (and it can happen even with purines and pyrimidines). My guess is that these amino acids were either replaced by more complex ones, or simply dispensed. But the question becomes more complex when we consider the coevolution of the adapters with the amino acid repertoire. Again, we find the chicken-and-egg problem: did the adapters evolve as a response to a wider choice of amino acids, or was it the variability during the evolution of the adapters that allowed more amino acids to be "invented" and included in a proto-code? I would rather leave these questions for the future, but we should not forget them!

*The simulated random codon assignments demonstrate that the rarer amino acids are better Stickland pairs, hence the increasing mean ATP yield. If so, why did Gly and Ala had remained in the code? This is only plausible if we assume that the protocells still relied on external amino acid sources, thus subpar, but abundant pairs need to be maintained as well. Which could only be true in the early days of the RNA world, however we have seen that such a metabolic system by necessity appeared late (the author also suggest that the code appeared late, se 1st paragraph of the "Relation to the abiotic and early metabolism" section). I see a contradiction here*.

There is no contradiction: at the earliest times, say at the hydrothermal vents, the autotrophic metabolism would suffice. Away from the vents (or in latent periods of activity), cells would rely on external sources of energy (including amino acids). But indeed, the code (as such, for translation) was established at the times of LUCA. At this point amino acid biosynthesis and the citric acid cycle would have readily evolved. The period in between is where external sources of amino acids would be required. As you suggest: more complex amino acids were, to some extent, selectively advantageous (because of both catalysis and fermentation). My guess is that alanine and glycine remained for two reasons: first, most amino acids are electron donors, and only few are acceptors, setting a pressure to maintain glycine. Furthermore, the efficiency and antiquity of its reactivity with glycine would be too costly to simply dispose of (recall that the assumption is that there were ribozymes catalysing such reaction). Second, they are the simplest and most abundant, so quantity balances quality. As a rule of thumb, if we consider the ratio of the yield of electron donors vs. electron acceptors in the Miller experiment we find that it is 831.4: 482.51 *μ*mol, respectively. If we consider only that of alanine and glycine, it is 790: 440 *μ*mol. In other words, they constitute more than 90% of the total substrate for the Stickland reaction. I must point that I did not take these proportions into account in the simulations; it remains to be studied how much the distribution of the amino acids in solution affect the yield of ATP. It is indeed a pertinent and relevant question.

*The author could have assessed how much better is the current genetic code compared to random codes in being accordance with the hypothesis. I know that there are novel amino acids, and also the code as we know it has been evolved to resist mutations and not to maintain Stickland pairs, but still it might be worth doing*.

Consider first the ATP yield of random codes that use only alanine, glycine, aspartic acid and valine. In Higgs' *four column theory *[50] the ancestral code assigns them to the codons NCN, NGN, NAN, and NUN, respectively. This code gives a yield of 0.073 mols of ATP. Compare this with a bootstrap using the same four amino acids, randomising the assignations: the mean yield of ATP is 1.06 ± 0.017 mols of ATP; the four column - four amino acid code gives a typical yield, as compared with the ensemble (*p *> 0.9). Since there is only one determiner base in the second position, it is an efficient code, given its complexity. A Higgs code that includes glutamic acid assigning it to codons NAR does not change its fermentation yield (because there are no new adapter pairs that bear Stickland reagents), whereas the mean yield of the ensemble is increased to 0.2 ± 0.028 mols of ATP. Naturally, the order of addition makes a difference. For instance, adding leucine before glutamic acid in the four column code, increases the yield ten-fold, to 0.104 mols of ATP (the ensemble is shifted to 0.2 ± 0.29 mols of ATP). Still, all these codes lie in the lower tails of the bootstrapped distributions, even though they are improved in every expansion. One interpretation is that expansions to the code were far more frequent than code rearrangements. The standard genetic code would yield 1.04 mols of ATP, a very typical value compared with the randomised ensemble, 1.01 ± 0.358 mols of ATP. The question is until when it is significant to add amino acids under the amino acid fermentation hypothesis. Clearly at some point this all breaks down. The fitness gained by adding an amino acid to the code necessarily involves costs, for it needs new ribozymes to ferment it, and scarce amino acids would require new and highly specific synthetases to attach them to the adapters.
